# A Review of the Potential Benefits of Plants Producing Berries in Skin Disorders

**DOI:** 10.3390/antiox9060542

**Published:** 2020-06-20

**Authors:** Stefano Piazza, Marco Fumagalli, Saba Khalilpour, Giulia Martinelli, Andrea Magnavacca, Mario Dell’Agli, Enrico Sangiovanni

**Affiliations:** 1 Department of Pharmacological and Biomolecular Sciences (DiSFeB), Università degli Studi di Milano, 20133 Milan, Italy; stefano.piazza@unimi.it (S.P.); marco.fumagalli3@unimi.it (M.F.); giulia.martinelli@unimi.it (G.M.); andrea.magnavacca@unimi.it (A.M.); 2 School of Medicine, Boston University, Arthritis Center/Rheumatology, Boston, MA 02118, USA; saba.khalilpour@unimi.it

**Keywords:** skin, inflammation, berry, UV, wounds, antioxidant, immunity

## Abstract

During the last 30 years, berries have gained great attention as functional food against several risk factors in chronic diseases. The number of related publications on Pubmed rose from 1000 items in 1990 to more than 11,000 in 2019. Despite the fact that a common and clear definition of “berries” is not shared among different scientific areas, the phytochemical pattern of these fruits is mainly characterized by anthocyanins, flavanols, flavonols, and tannins, which showed antioxidant and anti-inflammatory properties in humans. Skin insults, like wounds, UV rays, and excessive inflammatory responses, may lead to chronic dermatological disorders, conditions often characterized by long-term treatments. The application of berries for skin protection is sustained by long traditional use, but many observations still require a clear pharmacological validation. This review summarizes the scientific evidence, published on EMBASE, MEDLINE, and Scholar, to identify extraction methods, way of administration, dose, and mechanism of action of berries for potential dermatological treatments. Promising in vitro and in vivo evidence of *Punica granatum* L. and *Vitis vinifera* L. supports wound healing and photoprotection, while *Schisandra chinensis* (Turcz.) Baill. and *Vaccinium* spp. showed clear immunomodulatory effects. Oral or topical administrations of these berries justify the evaluation of new translational studies to validate their efficacy in humans.

## 1. Introduction

The skin is the largest organ in the human body and acts as a physical barrier to prevent the invasion of external pathogens. From a structural point of view, this organ is divided in two distinct layers—the epidermis, the outermost part, which is formed by differentiating layers of keratinocytes; and the dermis, characterized by fibroblasts and the production of extracellular matrix. Skin lesions are caused by several factors including chronic inflammation, due to infections or autoimmune diseases, UV radiation, and wounds. Cutaneous reactions are the result of interactions between different immune and non-immune cellular components [1]. Beside classical skin injuries, such as infections and wounds, chronic dermatitis is actually underlined as one of the main challenges of our time due to the relatively recent increase in prevalence all over the world and the necessity of new drugs to treat it. França and Lotti [2] introduced a manual of integrative dermatology including medicinal plants, with the purpose to support classical “single target*”* pharmacotherapy [3]. Several plant extracts are known as a promising source for skin care in different fields, in term of anti-inflammatory [4], photoprotective [5], and wound healing agents [6]. Fruits and leaves from different berries are often traditionally used for medicinal purposes, like inducing diuresis, diaphoresis, and, notably, disinfection and astringency in epithelial inflammations [7,8], suggesting among other things the interest of the byproducts of fruit production for biological studies [9,10].

According to several ethnopharmacological surveys, dermatological disorders are one of the main purposes for treatment by traditional practices [11,12]. By empirical trials, the antibacterial and anti-inflammatory properties of natural compounds have been selected and remain the main approach to treat skin afflictions. The review by Cavero et al. [12] identified blackcurrant, by frequency of use, as the second of the top 10 plants used to treat dermatological disorders in the Mediterranean area. Pomegranate has also been reviewed for the promotion of wound healing [13]; however, more studies are required before considering pomegranate extracts to be effective in other skin disorders. 

The purpose of this review is to summarize the potential skin benefits of berry-producing plants. To proceed in this work, we need to clarify the meaning of “berry,” for which a widely accepted definition is actually missing. Papers commonly refer to berries based on divergent definitions, mixing botanical and common plant names. The manual of ethnobotany from Schmidt and Klaser Cheng [14] categorizes berries as fruits with a soft fleshy pericarp, usually from a compound ovary with more than one seed or a simple ovary—for example, tomatoes (*Solanum lycopersicum* L.), and grapes (*Vitis* spp.). Some berries derive from inferior ovaries and have remnants of flower parts at their tip—for example, blueberries and cranberries (*Vaccinium* spp.), pomegranate (*Punica granatum* L.), and bananas (*Musa* spp.). 

Taking into account the botanical definition, the following review focuses mostly on plants producing small edible fruits with high organoleptic properties that are widespread especially in temperate areas and widely used both as food and/or for medicinal purposes. Pomegranate fruit is a leathery-skinned berry, which has been reviewed despite its size, and also strawberries, commonly but incorrectly considered in this category, represent a botanical exception, but both included in this research.

From a phytochemical point of view, berries typically share a common pattern of compounds characterized by flavonoids (mainly flavanols and flavonols) including anthocyanins, organic acids, and tannins. In addition to the previous compounds, there are some fruits characterized by a peculiar lipidic composition, as acaj (*Euterpe oleracea* Mart.) and black currants (*Ribes nigrum* L.), or lignans like schisandra (*Schisandra chinensis* (Turcz.) Baill.) berries [15,16,17].

This review examines the current scientific literature to collect the contribution of berries to skin health, highlighting the part of the plant used and the standardization of the active principles. 

## 2. Materials and Methods 

The literature research was conducted in January 2020 using the main database from the biomedical area (MEDLINE, EMBASE) and Google Scholar. No limit was applied for year of publication, but only papers in the English language have been considered. Research articles were searched for title and abstract using the following search terms: the words “berry” or “berries” or the Latin name or common name or vernacular name of the plant matched with skin, skin inflammation, dermatitis, psoriasis, burn, wound, UV, lymphocyte, CD4, keratinocytes, and fibroblasts.

The selected inclusion criteria regard the effect of berries against UV, cytokines, synthetic stimuli, allergens, and wounds, without any restriction on the way and form of administration. Papers concerning antibiotic or anti-cancer activities, as well as papers investigating combination of different plant extracts or purified compounds, were out of the scope of this review.

Works without information of doses or botanical name reference were also excluded. The protective effect of grapevine and pomegranate on particulate matter-induced inflammation have been recently reviewed [18] and were not considered. 

## 3. Results

The analysis of the literature identified plants belonging to the following 10 genera: *Euterpe*, *Fragaria*, *Lonicera*, *Punica*, *Ribes*, *Rubus*, *Sambucus*, *Schisandra*, *Vaccinium*, and *Vitis*. A list of the species investigated at cutaneous level is reported in Table 1. The research was divided by genus, in alphabetical order, and classified in three categories according to the investigated topic: skin inflammation and immunity, UV, and wound healing. The acronyms used in the results section are described in Table S1.

### 3.1. Euterpe

*Euterpe* (fam. *Arecaceae*) is a genus of palm tree that includes seven accepted species. *Euterpe oleracea* Mart. (açai) has been cultivated, especially in northern South America, for a wide range of health-promoting benefits due to its reportedly high levels of antioxidants. Unfortunately, until now, the supposed benefits have been poorly supported by scientific data [19]. The predominant chemical constituents of the fruit are flavonoids including anthocyanins, a small amount of lignans and a lipidic fraction (fatty acids, sterols, tocopherol) [20,21].

#### 3.1.1. Skin Inflammation and Immunity

One in vivo study tested the effects of *E. oleracea* on skin inflammation and immunity. The oil of açai fruits (commercially available) was evaluated at 1226.8 mg/kg by Favacho et al. in different in vivo models of skin inflammation. In a rat paw edema model, male albino Wistar rats (five per group) were treated orally with açai oil, indomethacin (10 mg/kg) or distilled water (0.5 mL) after carrageenan injection (30 min, 1000 μg/paw). Açai oil inhibited the edema formation over 6 h by 29.84% (*p* < 0.01) compared to the negative control, but less compared to indomethacin. Animals treated daily (six consecutive days) with açai oil had a 36.66% reduction of granulomatous tissue formation (*p* < 0.01) compared to the control group, more than dexamethasone (0.2 mg/kg). Finally, cutaneous inflammation was induced in male albino Swiss mice with croton oil solution (1 mg/ear) for 30 min. The topical application of açai oil and dexamethasone (0.5 mg/kg) reduced inflammation by 37.9% and 68.8%, respectively [22].

#### 3.1.2. UV Damage

One in vitro study investigated the activity of *E. oleracea *on UV-induced skin injuries. Petruk et al. tested the antioxidant effects of methanol/water extract from açai fruits (10 mg/mL) in BALB/3T3 fibroblasts exposed to UVA (100 J/cm^2^). Pretreatment with the extract significantly protected cells from UVA-induced oxidative stress through inhibition of ROS production, lipid peroxidation, and intracellular glutathione depletion (GSH form). Moreover, açai extract significantly reduced the phosphorylation levels of p38, MAPKAPK-2 and HSP-27 (proteins related to oxidative stress). Among the extract compounds, the authors identified malvidin and cyanidin derivatives as the most active molecules, able to counteract the negative effects induced by UVA irradiation [20].

#### 3.1.3. Wound Healing

Two studies (one in vivo and one in vitro) investigated the effects of açai berry on skin wound healing. A water extract, prepared by Kang et al., was evaluated in vivo in a skin excision wound model (six-week-old male Sprague Dawley rats). As positive control group, an ointment containing 20 mg/g (2%) of sodium fusidate was applied, while three groups were topically treated with 1%, 3% or 5% açai extract, for 18 days. The treatment showed macroscopic and histopathological beneficial effects, supported by the increased gene expression of type I collagen, VEGF, and fibronectin. MMP-1 and IL-1β mRNA was decreased by açai extract [23]. The same preparation (1 mg/mL) increased the number of fibroblasts (HS68 cells), cultured for 48 h and 72 h, enhancing cell migration to scratched area, upregulating fibronectin, and downregulating MMP-1 mRNA expression [23].

### 3.2. Fragaria

There are 17 accepted species of *Fragaria* (fam. *Rosaceae*) that widely grow in all temperate regions of the world. *Fragaria* × *ananassa* (Duchesne ex Weston) Duchesne ex Rozier is a hybrid species whose fruits, collectively known as the common strawberries, are rich in bioactive compounds such as flavonoids (including anthocyanins), proanthocyanidins, and phenolic acids [24,25]. Scientific studies provide evidence of the anti-inflammatory and protective effects of strawberries in postprandial hyperglycemia and metabolic syndrome [26,27].

#### 3.2.1. Skin Inflammation and Immunity

The anti-inflammatory effect of strawberry (*F.* × *ananassa*) at skin level was investigated in two in vitro studies. Li et al. tested the methanol extract from fruits, fractionated as aqueous (polar) and dichlorometane (apolar) fractions [28]. JB6 Cl 41 (epidermal mouse cells), were elicited by benzoapyrene-diol-epoxide (BaPDE) to simulate inflammatory and cancerogenic environment. Neither strawberry polar or apolar fractions (10-50 µg/mL) inhibited the NF-κB or AP-1 driven transcription or VEGF promoter activity, following BaPDE induction. The polar fraction (25 µg/mL) did not impair PI3K activity, Akt (p70), and MAPK (ERK, JNK, p38) phosphorylation but inhibited NFAT and TNF-α promoter activity by 50%, whereas no effect was shown with apolar fraction, thus suggesting the major relevance of polyphenols.

Gasparrini et al. [29] demonstrated the anti-inflammatory activity of methanol extract from strawberry fruit (“Alba”) in human fibroblasts (HDF) at 50–100 µg/mL. After challenge with lipopolysaccharide (LPS), the extract inhibited intracellular ROS formation in a concentration-dependent fashion. Conversely, at both the doses, 50% reduction of necrosis and apoptosis was observed. LPS-induced pro-inflammatory mediators, including TNF-α, IL-1β, IL-6, and p-IKBα (−50%), were impaired by the extract (100 µg/mL), while anti-inflammatory IL-10 was increased (2 folds vs. ctrl). The mechanism was attributed to pro-oxidant and inflammatory pathways impairment, since strawberry extract increased catalase and superoxide dismutase (SOD) expression via Nrf-2 activation (50–100 µg/mL, without LPS) and marginally increased p-AMPK.

#### 3.2.2. UV Damage

The photoprotective effect of *F.* × *ananassa* at skin level was investigated by three in vitro studies from the same research group. Giampieri et al. [30] evaluated the effect of a methanol extract (0.05, 0.25, and 0.5 mg/mL) from strawberry (“Sveva”) on primary cultures of human dermal fibroblasts, irradiated with UVA (275 kJ/m^2^, or 27,5 J/cm^2^) at maximum 30 min. Pre-treatment with 0.5 mg/mL led to higher survival rate (MTT test) after 5 or 15 min of exposure, while, at 30 min, the differences were not significant. Cells pre-treated with 0.25 mg/mL and 0.5 mg/mL showed a significant decrease of the UVA-induced DNA damage.

Gasparrini et al. [31] included different concentrations of methanolic strawberry extract (0.025–1 mg/mL, cultivar “Alba”),reduced CoQ10 (25–100 μg/mL) or oxidized CoQ10 (25–100 μg/mL), alone or combined with a SPF10 sun protective formulation, not in contact with cells but spread onto quartz-bottom petri dishes on top of the wells. The formulations containing higher concentrations of strawberry extract obtained a significantly greater viability of UVA-exposed (27.5 J/cm^2^) primary human fibroblasts (up to 28% for the highest concentration of strawberry) compared to UVA condition. Similar trends were observed for the formulations prepared with CoQ10, both reduced and oxidized. Combination of higher concentrations of strawberry extract, CoQox or CoQred with a SPF10 formulation increased cell viability, but the maximum effect was obtained when extract was combined with CoQox and CoQred.

In a similar setting, the same group evaluated SFP10 formulation (2 mg/cm^2^), containing strawberry extract (50 μg/mL) or reduced CoQ10 (50 μg/mL) or the mix of them, measuring different parameters [32]. The combined action of strawberry and CoQ10red significantly reduced the level of dead cells compared to UV-exposed cells, but no significant differences were detected in apoptosis rate between the tested groups, unlike other works on berries extracts [33,34]. Strawberry and CoQ10red combination improved mitochondrial functionality (increasing the OCR value) and reduced ROS production and the levels of inflammatory biomarkers (NF-κB, p-IκBα, TNF-α, IL-6, and IL-1β), whose expression considerably increased after UV-exposure. In addition, the combination of extract and CoQ10red increased Nrf-2, SOD, catalase, and HO-1 levels more efficiently compared to other formulations.

### 3.3. Lonicera

There are 103 species of vines and shrubs accepted in the *Lonicera* genus (fam. *Caprifoliaceae*) and distributed throughout the northern hemisphere, but only *Lonicera caerulea* L. (honeyberry) has been studied at the cutaneous level. The fruits are a rich source of phenolic compound and anthocyanins, able to regulate the microvascular tissue, supporting the treatment of various eye disorders, with a potential application against metabolic and cardiovascular diseases.

#### UV Damage

Two in vitro studies tested the effects of *L. caerulea* on UV-induced skin damage. Svobodova et al. investigated the photoprotective activity of the phenolic fraction from *L. caerulea* fruits on UVA-induced oxidative stress in human keratinocytes (HaCaT cells). The fruits were percolated with aqueous solution of H_3_PO_4_ (0.1%; *v*/*v*), and the obtained extract was purified on a column filled with non-ionic polystyrene-divinylbenzene resin; the adsorbed organic compounds were desorbed by ethanol. The phenolic fraction (0.4% of fresh fruits) contained 20.1% of phenols including 18.5% anthocyanins (cyanidin-3-*O*-glucoside was the most abundant). The cells were pre-treated (1h) with the phenolic fraction (1-250 mg/L) before UVA irradiation (200 mJ/cm^2^) or after UVA exposure (for 4 h); doses of UVA rays were 10–30 J/cm^2^. Pre-treatment showed a significant reduction of UVA-induced ROS production at 50–100–250 mg/L, while post-treatment showed the maximal reduction at 50 mg/L (−72%). Phenolic fraction from *L. caerulea* was also able to suppress lipid peroxidation. Pre-treatment increased glutathione levels, especially at 2.5 and 5 mg/L, while post-treatment showed the maximal effect at 100 mg/L [35].

Svobododa et al. evaluated the effects of another phenolic fraction of *L. caerulea* (characterized by 77% of (*w*/*w*) anthocyanins, cyanidin-3-*O*-glucoside was the most abundant) against UVB-induced damages in HaCaT cells. The cells were pre-treated (1 h) with the phenolic fraction (5–50 mg/L) before UVB irradiation (200 mJ/cm^2^) or treated (4/8 h) after UVB exposure. The extract was able to reduce DNA single strand breaks and caspase-3 and caspase-9 activity induced by UVB irradiation during both pre- and post-treatment. DNA fragmentation, an index of apoptotic process, was prevented, by *L. caerulea* extract (25 mg/L), while post-treatment was efficient at all concentrations tested. The authors demonstrated that this extract decreased reactive oxygen and nitrogen species (RONS) production in keratinocytes, and pre-treatment reached 50% of inhibition at the highest concentration tested whereas post-treatment showed a concentration dependent effect. The extract also showed the ability to decrease UVB-induced IL-6 productio, in pre-treatment (25 mg/L) and post-treatment (10 mg/L and 25 mg/L) [36].

### 3.4. Punica

*Punica* (fam. *Lythraceae*) is a genus consisting of only two species (*Punica granatum* L. and *Punica protopunica* Balf. f.) and characterized by fruit-bearing deciduous shrubs or small trees. The most studied plant, pomegranate (*Punica granatum* L.), is widely distributed in northern India and in the Mediterranean region. The various biological activities are attributed to the fruits (and their seeds), due to the presence of a variety of phytochemicals including hydrolysable (ellagitannins) and condensed tannins, ellagic acid, punicic acid, and flavonoids (mostly anthocyanins) [37].

#### 3.4.1. Skin Inflammation and Immunity

Despite the number of publications on the anti-inflammatory effects of *P. granatum* in other epithelial cells (for this purpose, activity at gastro-intestinal level was described by Colombo et al. [38]), the effects of pomegranate have never been reviewed in skin cells, such as keratinocytes, fibroblasts, and dendritic cells or in Th1/Th2 lymphocytes and related sub-families—namely, cells with crucial role in atopic dermatitis and psoriasis [39,40].

Lee et al. (2008) screened the effect of 1400 plant extracts on the NFAT-driven transcription challenged by PMA in stably transfected line of lymphoblasts (Jurkat cells). Since acetone extract from pomegranate fruit showed a great inhibitory activity, one of its main ellagitannins, punicalagin, was investigated showing an IC_50_ of 15 µM. At lower concentrations, punicalagin inhibited IL-2 release and mRNA transcription (IC_50_ < 5 µM), while higher concentrations impaired Jurkat proliferation (–56% at 40 µM). Punicalagin was further injected i.p. (5–10 mg/kg) in mice after the induction of ear edema by PMA. The treatment (10 mg/kg) halved the swelling with a parallel strong reduction of CD3+ lymphocytes infiltration in the tissue. 

Another ex vivo study revealed the anti-inflammatory effect of pomegranate extract and the trans-epidermal passage of punicalagin [41]. A rind extract standardized in punicalagin (20%), prepared by infusion, was applied topically (100 µg/mL) to porcine skin in Franz cell model. The effect was compared with a tannin-enriched extract (100 µg/mL) obtained by resin exclusion from the first (84% punicalagin). The crude rind extracts inhibited COX-2 more potently than tannin-enriched extract (−70% and −40%, respectively). Moreover, the recovery of punicalagin in the basal layer of skin after stripping test was 2.39 ± 0.29 nM/cm^2^. Despite the nM range is far from the concentrations mentioned in other papers included herein, our group recently demonstrated that ellagitannins from *Rubus* spp. inhibited IL-8 release in gastric epithelial cells, with IC_50_ of 570–580 nM [42]. 

#### 3.4.2. UV Damage

*P. granatum* fruit is widely recognized as a source of phytochemicals with high antioxidant activity. In 2011, Afaq et al. have reviewed the literature on photoprotective effect of pomegranate extract, collecting five papers. From 2011 until 2020, 12 papers were published—seven in vitro, three in vivo, one ex vivo*,* and one in humans. 

Afaq et al. [43] examined the effect of pomegranate fruit-derived products on reconstituted human epidermis challenged by UVB (60 mJ/cm^2^). The juice (1–2 μL/0.1 mL/well), the extract (5–10 μg/0.1 mL/well), and the oil (1–2 μL/0.1 mL/well) from fruit inhibited cyclobutane pyrimidine dimers (CPD), 8-Oxo-2’-deoxyguanosine (8-oxodG), and protein oxidation and proliferating cell nuclear antigen (PCNA) protein expression. Moreover, all the products inhibited UVB-induced collagenase (MMP-1), gelatinases (MMP-2, MMP-9), stromelysin (MMP-3), marilysin (MMP-7), elastase (MMP-12), and tropoelastin. The activity of MMP-2 and MMP-9 was reduced as well. The authors attributed the effect on metalloproteases to c-jun and c-fos expression. Similarly, in a previous in vitro study, Afaq et al. [44] demonstrated the effect of a commercial pomegranate extract (10, 20, and 40 μg/mL) on NHEK (normal human epidermal keratinocytes) challenged by 40 mJ/cm^2^ UVB. The treatment inhibited, in a concentration-dependent way, the phosphorylation of ERK1/2, JNK1/2, and p38 after UVB exposure. The downstream phosphorylation of kinase belonging to NF-κB pathway and translocation of p65 were lowered in a concentration-dependent fashion. 

The fruit extract (edible part extracted by acetone:water 70:30) 0.2% *w*/*v* was also administrated in vivo through drinking water to SKH-1 mice before the irradiation with UVB (180 mJ/kg). Skin biopsies revealed the impairment of COX-2 and NOS expression and MAPKs, STAT3, and NF-κB/p65 phosphorylation. The antioxidant effect was related to the reduction of adducts like 8-Oxo-2’-deoxyguanosine (8-oxodG 44) and H_2_O_2_ [45,46].

Another in vivo study from the same research group investigated the effect of the edible fruit extract in SKH-1 mice using the same experimental conditions, but multiple UVB exposure. Oral administration of the extract inhibited hyperplasia, infiltration of leukocytes, protein oxidation and lipoperoxidation. Moreover, cyclooxygenase-2 (COX-2) and inducible nitric oxide synthase (iNOS) protein expression were impaired. The anti-proliferative effect was deduced by the reduction of cyclin D1 and PCNA in mouse epidermis. The inhibition of MMPs (MMP-2, MMP-3, and MMP-9) and AP-1 protein expression was also verified in the context of multiple exposure [47]. 

Accordingly, Syed et al. [48] carried out experiments using the acetone:water (70:30) extract from the squeezed juice of pomegranate, but NHEK cells were challenged by UVA (4 J/cm^2^). Pre-treatment with 60–100 μg/mL of extract before exposure resulted in a concentration-dependent inhibition of the STAT3, Akt and ERK1/2 phosphorylation. Moreover, it was also found that pomegranate inhibited UVA-induced mTOR and increased the cell-cycle arrest in the G1 phase leading to apoptosis.

Regarding specific anti-oxidant mechanisms, Zaid et al. [49] evaluated the effect of a concentrated juice from pomegranate on UVB (15–30 mJ/cm^2^)-challenged keratinocytes (HaCaT), measuring recovered glutathione (GSH form) and lipid peroxidation levels. As previously reported, pomegranate was also able to restore MMPs/TIMPs unbalance, and the molecular mechanism was attributed to the impairment of c-Jun and MAPKs phosphorylation.

The edible portion of the fruit, in comparison with peel and seeds, was tested after methanol extraction also by Park et al. [50], showing the effect on primary fibroblasts exposed to UVB (170 mJ/cm^2^). Treated cells (3–10 µg/mL of each extract) expressed higher concentration of procollagen type I than those irradiated without pomegranate treatment: this effect was not specific to any particular part of the plant, although the edible part showed the weakest effect. Once again, the extracts were able to inhibit MMP-1 expression. The authors attributed the observed effect to (+)−catechin, but, as it often happens, the effect of the crude extracts was greater than the individual compound.

Baccarin et al. [51] investigated the effect of *P. granatum* seed oil or medium chain trygliceride nanoemulsion entrapping a pomegranate peel polyphenol-rich ethyl acetate fraction. Both free polyphenolic fraction (20 and 50 μg/mL) and nanoemulsions were capable to protect the human keratinocyte HaCaT cells from UVB-induced (90 mJ/cm^2^) DNA damage and restore cells viability. There was no statistically significant difference between free fraction and nanoemulsions. According to the photoprotection results, 50 μg/mL reduced the IL-8 release in irradiated cells. 

#### 3.4.3. Wound Healing

Pomegranate is the most investigated berry for wound healing. The search selected 15 papers—13 of them showed in vivo evidence, one in vitro, and one in humans. This section describes the most representative studies; for a more extensive revision of papers and details, refer to Table S2. 

The most frequently exploited plant material for In Vivo studies was the peel from fruit, extracted by maceration with combined polar solvents like ethanol:water or methanol:water, namely more organic solvent than water (at least a ratio of 70:30). Lukiswanto et al. [52] and Yuniarti et al. [53] obtained the healing activity, respectively, in the models of hot plate-induced burn and linear incision, with similar concentrations of corresponding ellagic acid (4% and 3% of the ointment, about 20–15 mg/kg referring to 0.5 g of topical application). Mo et al. [54] paralleled the effect of pomegranate peel extract (5%) and the relative amount of pure ellagic acid (0.65%, close to 3.25 mg/kg) on excision, incision, and burn model. Despite the lower amount of ellagic acid, the authors clearly demonstrated that the individual compound is responsible, at least in part, for the premature healing versus control. The mechanism of action was partially explained—despite the analogue use of whole fruit extracts, standardized on ellagic acid amount (40%), Lukiswanto et al. [52] and Yuniarti et al. [53] observed the increase in collagen deposition but also divergent effects on polymorphonuclear (PMN) cells infiltration and angiogenesis. However, Mo et al. [54] attributed the increase in hydroxyproline content and collagen deposition to the extract, but not to ellagic acid; granulation and inflammation were absent in the treated tissue, even when ellagic acid was used alone, thus supporting the observations of Lukiswanto et al. [52] about impaired angiogenesis and PMN infiltration. In addition to the anti-inflammatory activity, ellagic acid (0.65%) counteracts the expression of myeloperoxidase in damaged tissue (−53%), leading to antioxidant activity [54]. Hayouni et al. [55] obtained similar results in guinea pig wound excision model using an extract from peel, rich in ellagic acid, punicalagin, and anthocyanins. Ellagic acid was recorded as the major relative peak. The 5% ointment enhanced the healing by collagen deposition and fibroblasts proliferation, while impaired leucocytes infiltration. 

Two papers [56,57] explored the in vivo healing effect of flowers extract. Polyphenols standardization or qualitative characterization were absent; interestingly the latter used a very low dose—0.2% ointment (corresponding to about 2 mg/kg). The demonstration of wound contraction was not supported by additional experiments aimed to elucidate the mechanism of action. 

A single in vitro study by Aslam et al. [58] reported that the peel extract stimulated fibroblast migration and pro-collagen I and MMP-1 release in vitro, while oil from seeds was able to enhance keratinocytes migration. The concentration was in the order of μL/mL of extract without any titration and there was no comparison with positive control.

One case-report corroborates the possible topical application of pomegranate peel extract for wound healing [59]. A woman with non-responsive chronic wound, 76 years old, with no other concomitant pathologies but a history of vascular disease, was treated successfully with a 2% hydrogel for 12 weeks, till the complete remission. The hydroalcoholic extract from pomegranate peel was standardized in punicalagin (plausibly mix of isomers A and B) and punicalin, which should represent, according to the authors, not less than 0.49% of the formulation. 

### 3.5. Ribes

The *Ribes* genus (fam. *Grossulariaceae*) consists of 194 species, which are widespread in the temperate regions of the Northern Hemisphere. *Ribes nigrum* L. is the *Ribes* species with the highest number of papers in the literature, showing potential to improve regulation of blood glucose and disease associate inflammation, thanks to the high levels of anthocyanins and proanthocyanidins [60]. *Ribes fasciculatum* var. *chinense* Maxim. received less attention, although it has been traditionally used in Korea to treat inflammatory diseases.

#### 3.5.1. Skin Inflammation and Immunity

Two in vivo studies investigated the activity of different species of *Ribes* on skin inflammation and immunity. Mihele et al. investigated the healing activity of a preparation based on *R. nigrum* essential oil. The essential oil was incorporated in a water-soluble gel base at the concentration of 5%. Male Wistar rats were divided in different groups, which were treated as follows (one daily topical application for 12 days)—a control group, a group treated with gel base alone or with *R. nigrum* essential oil (5%), and a group treated with cicatrizin as reference compound). The wound was induced in rats by burning with a metallic disk device. The results showed that the preparation containing 5% of *R. nigrum* essential oil promoted the healing of induced burn wounds, and its effect was similar to that exerted by reference compound [61].

In another paper, the authors describe the in vivo wound healing effects of methanol extracts from the leaves of different *Ribes* species (*R. alpinum*, *R. anatolica*, *R. multiflorum*, *R. nigrum*, *R. orientale*, *R. petraeum*, *R. rubrum*, and *R. uva-crispa*). Each extract was prepared in 1% concentration in the base ointment. The dorsal parts of male Swiss albino mice were subjected to incision and excision wounds; the treatment was applied once daily on the incised wounds for 10 days and excised wounds for 15 days. *R. nigrum* and *R. multiflorum* extracts showed the most promising results, the preparations significantly increased wound breaking strength and significantly reduced the wound areas on the 15th day. Since *R. nigrum* was found to be the most active species, the authors further investigated the wound healing potential of the fractions obtained by the methanol extract of *R. nigrum* leaves, discovering the ethyl acetate as the most active [62].

#### 3.5.2. UV Damage

One in vivo study evaluated the effects of a polysaccharide from *R. nigrum* on UV-induced skin damage. The same research group also investigated the effects of a polysaccharide from *R. nigrum* (named cassis polysaccharide) in hairless mice irradiated with UV light (90 mJ/cm^2^). Mice were divided in different groups as follows: two groups (control and irradiation) in which mice were fed with a standard diet (called AIN-93G); two other groups were fed a diet of AIN-93G containing either 0.2% or 1% cassis polysaccharide powder. After 2 weeks, mice from irradiation group and both the cassis polysaccharide groups were exposed to UV light (90 mJ/cm^2^). The results demonstrated that the polysaccharide was able to counteract skin dehydration induced by UV irradiation; the stratum corneum hydration was significantly higher in both cassis polysaccharide groups (UV-treated groups) than in the UV-treated control group. Compared to the UV control group, IL-6 gene expression level was significantly lower in both cassis polysaccharide groups (UV treated), while the MMP-13 gene expression level was significantly lower only in 1% cassis polysaccharide group [63].

#### 3.5.3. Wound Healing

According to literature, two in vivo studies investigated the effects of *Ribes* genus on skin wound healing. Jung et al. evaluated the activity of an extract (70% ethanol) obtained from the roots of *R. fasciculatum* on in vivo model of atopic dermatitis (2,4-dinitrochlorobenzene, DNCB)-induced atopic dermatitis in mice). Male BALB/c mice were sensitized with 0.15% DNCB applied to the dorsal skin twice per week for five weeks; after three weeks, *R. fasciculatum* (200 mg/kg) was orally administered two weeks until the end of the experiment. Treatment of animals with *R. fasciculatum* extract significantly reduced the atopic dermatitis and IgE levels in serum induced by the application of DNCB [64]. 

Ashigai et al. studied the effects of the administration of cassis polysaccharide, extracted from *R. nigrum*, on atopic dermatitis in NC/Nga mice. Male NC/Nga mice, that develop symptoms of atopic dermatitis, received cassis polysaccharide which was dissolved in distilled water and orally administered once a day throughout the experimental period at a dose of 12 or 60 mg/kg. The atopic dermatitis scores of the animals treated with the lower dose of cassis polysaccharide were significantly lower than those of the control group at 21 and 28 days, while the atopic scores of the animals treated with the highest dose of cassis polysaccharide were significantly lower than those of the control starting at 14 days. Moreover, the serum IgE level in the animals treated with cassis polysaccharide at 60 mg/kg was significantly lower compared with the control group. The animals receiving the highest dose of cassis polysaccharide showed a significantly decreased number of mast cells in the epidermis [65].

### 3.6. Rubus

Blackberries (*Rubus fruticosus* L.) and raspberries (*Rubus idaeus* L.) are the most popular fruits belonging to the *Rubus* genus (fam. Rosaceae), which consists of hundreds of species. *Rubus fruticosus* L. leaves are traditionally used to treat diarrhea due to their high tannins content. Fruits have a considerable number of anthocyanins, which confer the typical color to the fruit, and ellagitannins. In addition to the previous two species, other cutaneous studies have highlighted the potential use of *Rubus adenotrichus* Schltdl. (tropical highland blackberry), *Rubus imperialis* Cham. & Schltdl., and *Rubus occidentalis* L. (black raspberry). 

#### 3.6.1. Skin Inflammation and Immunity

According to the wound healing section, the literature was revised for *R. adenotrichus*, *R. fruticosus*, *R. occidentalis*, *R. idaeus*, and *R. imperialis*, and just one in vitro and one in vivo paper were found.

In addition to experiments on wound healing, Tonin et al. [66] evaluated the anti-inflammatory activity of *R. imperialis* methanol extract from aerial parts. The extract decreased nitric oxide release by neutrophils from BALB/c mice, inflamed by intraperitoneal injection of oyster glycogen. The triterpenoid saponin niga-ichigoside F1 (10 μM) identified in *Rubus* spp. extract exerted the same effect. In Swiss mice pouch model, after topical treatment with the extract (100 mg/kg), the infiltration of neutrophils induced by LPS was comparable to the reference compound indomethacin (30 mg/kg).

Li et al. [28] treated JB6 Cl 41 cells (epidermal mouse cells) with *R. occidentalis* methanol extract from fruit as well as strawberry, previously described. Cells were elicited by BaPDE (benzoapyrene-diol-epoxide), a marker of pyrenes exposure, which exhibits pro-inflammatory and carcinogenic effects. Polar and apolar fractions were divided by water and dichloromethane extraction, respectively: only the pre-treatment with the first fraction (25 μg/mL) totally inhibited the NF-κB or AP-1 driven transcription. Similarly, polar fraction completely inhibited NFAT and inhibited dichloromethane fraction by 50%. Again, the polar fraction inhibited TNF-α and VEGF promoter activity at the highest concentration of 50 μg/mL. Additional mechanisms at upstream level of NF-κB or AP-1 activation were purposed—the polar fraction also inhibited PI-3K activity and the phosphorylation of Akt, p70, and MAPKs (ERK, JNK, p38). 

#### 3.6.2. UV Damage

The revision on this topic identified two papers reporting in vivo and two papers reporting in vitro evidence. Among the *Rubus* spp., *R. adenotrichus*, *R. fruticosus,* and *R. occidentalis* have been previously investigated. 

Divya et al. [67] treated topically SKH-1 hairless mice with blackberry extract (10% and 20% in acetone, 50 μL) after dorsal UVB multiple exposure (100 mJ/cm^2^, three times/week for 10 weeks). As highlighted by the histological exam, cutaneous edema, hyperplasia, and leukocyte infiltration were inhibited. In parallel, the antioxidant activity was explained by the decrease of myeloperoxidase (MPO) activity, DNA damage (8-oxodG and CPD formation), malondialdehyde (MDA) levels, and glutathione depletion. Furthermore, the extract impaired inflammatory mediators such as iNOS, COX-2, PGE2, TNF-α, and IL-6 expression. Accordingly, molecular mechanisms involved the inhibition of ERK1/2, p38, JNK1/2, and MKK4 phosphorylation. At the transcriptional level, the inhibition of pro-inflammatory mediators was ascribed to the NF-κB pathway impairment. Finally, the extract was also able to inhibit the perturbing effect of UVB on cell cycle, lowering cyclin D1 and PCNA expression in skin.

Duncan et al. [68] evaluated the in vivo effect of *R. occidentalis* extract focusing on hexane fraction, further extracted by ethanol/H_2_O (80/20). Female SKH-1 hairless mice were exposed to one minimal erythemal dose of UVB (224 mJ/cm^2^) and treated with 500 μg of extract dissolved in 100 μL of vehicle applied topically against UV-mediated carcinogenesis (long follow-up) and inflammation (48 h follow-up). The total tumors number, size, and progression were drastically inhibited, correlating with significantly lower CD3+ foxp3+, but not CD8+ T cells in the tissue. Once again, the extract reduced oedema formation and DNA damage through antioxidant activity, related to dampened neutrophil activation (MPO), reduced p53-postive cells, and ROS-derived DNA adducts (8-oxodG). 

Blackberry (*R. fruticosus*, ‘Hull Thornless’) was also investigated in primary keratinocytes from the epidermis of neonatal C57Bl/6 mice by Murapa et al. [69]. Anthocyanin-rich methanol fractions derived from either blackberry powder (100–200 μg/mL) or puree (50–80 μg/mL) were tested against UVB irradiation (100 mJ/cm^2^). In agreement with the previous authors, the methanol fraction of puree exhibited antioxidant effect (comparable to N-acetylcysteine 5 μM) through the enhancement of antioxidant enzymes (CAT, MnSOD, Gsta1, Gpx1/2).

Calvo-Castro et al. [34] observed a similar photoprotective effect in keratinocytes, treated with *R. adenotrichus* juice, directly diluted in medium (1:300 and 1:500), before or after UVB exposure (25 mJ/cm^2^). The pre-treatment (2 h) and post-treatment (24 h) with juice of NHEKs reduced UVB-mediated CPDs and 8-oxodG formation. Furthermore, the juice seemed to counteract DNA damage also by increasing apoptosis of damaged cells via activation of caspases 3, 8, and 9. CPDs reduction was confirmed in a 3D skin equivalent model of epidermal keratinocytes.

#### 3.6.3. Wound Healing

The revision of the literature included *R. adenotrichus*, *R. fruticosus*, *R. occidentalis*, *R. idaeus*, and *R. imperialis*. Only the last two species have been previously investigated for wound healing effect through two studies. Despite the small number of studies, preliminary evidence of In Vitro, In Vivo and human efficacy was provided. 

Tito et al. [70] carried out In Vitro experiments (keratinocytes HaCaT and fibroblasts HDF) on the liposoluble fraction of cultured cells from raspberry leaves, enriched in linoleic and alpha-linolenic acid. The addiction of 0.1% of liposoluble fraction to keratinocyte media determined the increase of beta-glucocerebrosidase expression and enzymatic activity in comparable manner to the LXR agonist TO-901317 (10 μM). Moreover, the expression level of genes involved in moisturization, like aquaporin 3, filaggrin, and involucrin was increased by 32%, 21%, and 75%, respectively. Markers of hyaluronic acid synthesis were increased in both cell lines. The expression of elastin, fibrillin I and lysil-oxidase, pro-collagen I, pro-collagen III, and fibronectin was enhanced in fibroblasts. The same authors extended the evidence to a pilot human study on 20 women with dry or very dry skin, in which the liposoluble extract cream increased (+20%) hydration versus moisturizing base cream.

On the other end, Tonin at al. [66] evaluated the effect of aerial parts (leaves and branches) of *R. imperialis*, extracted by methanol maceration. In analogy with mentioned inflammation experiments, the biological activity at 1–100 μg/mL was compared with triterpenoid saponin niga-ichigoside F1 (1–100 μM). *R. imperialis* (10 μg/mL), but not niga-ichigoside (100 μM), increased the migration of L929 fibroblasts. Since niga-ichigoside was not effective on fibroblast migration, only *R. imperialis* extract was selected for the in vivo experiment of wound excision (BALB/c mice): doses of 1–2.5% of extract in semisolid base determined an earlier reduction of wound area in comparison to control.

### 3.7. Sambucus

The genus of *Sambucus* (fam. *Adoxaceae*) is mainly distributed in the northern hemisphere, from temperate to subtropical regions, and consists of 22 species. The most commonly studied species are *Sambucus nigra* L. (elderberry) and *Sambucus ebulus* L., both characterized by a long tradition of use and, nowadays, investigated for different biological activities such as antibacterial, antidepressant, anticancer, and lipid lowering effects. Fruits and flowers of *S. nigra* L. are used as remedies against cold and flu.

#### 3.7.1. UV Damage

Only one in vitro study concerning the activity of *Sambucus* genus on UV-induced skin injuries was found in literature. 

Dried fruits of *S. nigra* were extracted with 70% ethanol; the extract was tested on human keratinocytes (HaCaT) exposed to UVB (125 mJ/cm^2^) at 1, 10, or 100 μg/mL. HaCaT cells were irradiated and then treated with the extract for 24h.

A concentration-dependent reduction of the UVB-induced reactive oxygen species release was observed. The extract (at 1 μg/mL) significantly inhibited UVB-induced MMP-1 secretion by 37.8%, reaching −48.0% at 100 μg/mL); moreover, the extract significantly decreased the release of IL-6 and VEGF by 40.4% and 35.5% at 100 μg/mL, respectively. The extract reduced UVB-induced MMP-1 gene expression by 28.6% at 100 μg/mL (*p* < 0.05) and significantly increased procollagen type I mRNA levels (by 162.7% at 100 μg/mL), which were downregulated by UVB irradiation. These effects were exerted through the inhibition of mitogen-activated protein kinases/activator protein-1 (MAPK/AP-1) and NF-κB signaling pathways; moreover, the extract blocked the extracellular matrix degradation in UVB-irradiated HaCaT cells. *S. nigra* extract increased the expression of Nrf2 and concentration-dependently enhanced HO-1 expression, in UVB irradiated cells; furthermore, the extract induced the translocation of Nrf2 from the cytoplasm to the nucleus [71].

#### 3.7.2. Wound Healing

In the literature, four papers highlighted the effects of *Sambucus* genus on wound healing in in vivo models.

Mogosanu et al., starting from the leaves of *S. nigra*, prepared a tincture by percolation (according to the Romanian Pharmacopoeia 10th edition) using a ratio of 1:5 plant material–extraction solvent (70° ethanol). The tincture was slowly evaporated obtaining a soft extract, which was mixed in a cold-cream H/L-type ointment base (according to United States Pharmacopoeia), in order to obtain a cold-cream with 10% soft extract. In this study, the authors used three groups of adult male Wistar rats (ten animals per group). Skin burns, of each group, were treated as follows: cold-cream with 10% *S. nigra leaf* soft extract for the first group, 1% silver sulfadiazine cream for the second group (reference group), cold-cream base for the third group (control group). The preparation was applied once a day. Rats treated with cold cream containing 10% soft extract experienced a positive evolution with a good epithelization, the wound healing was almost complete after 21 days, while the two other groups (1% silver sulfadiazine cream group and cold-cream base group) showed an incomplete epithelization after 21 days. In the group treated with cold-cream containing 10% soft extract, at 21 days after burn, the inflammatory infiltrate was considerably reduced, the granulation tissue was largely well recovered, and the re-epithelization of skin wound was well induced compared to reference and control group, in which the authors observed the persistence of a coagulation necrotic area and abundant inflammatory infiltrate [72].

Ghabacee et al. evaluated the wound healing capacity of the methanol extract obtained from *S. ebulus* fruits, using male Wistar albino rats for full-thickness wound. The extract was mixed with an ointment base to prepare a 5% ointment preparation. After the full-thickness wound, the animals were divided into three groups: control group, ointment base treated group and 5% ointment preparation treated group. The preparation was topically applied on the wound area, once a day for 12 days. At the end of the treatment period, in the group treated with the extract the wound contraction was faster compared to the other groups; moreover, the epidermal layer thickness (105.12 ± 4.38 μm), compared with that of the control group and ointment base treated group (78.09 ± 3.8 and 81.53 ± 4.1 μm, respectively), was significantly increased. In addition, the extract reduced the number of inflammatory cells, increased wound-healing rate, increased the organization of the granulation tissue formation and significantly improved collagen formation compared to the control groups [73].

Babaei et al. prepared an extract (70% methanol as solvent) using the root of *S. ebulus*, then, the extract was incorporated with Eucerin as the ointment base for the preparation of 2% or 5% topical ointments. These preparations were topically applied to animals (male Wistar rats) subjected to a full-thickness wound, once a day for 21 days. At the end of the experiment, the group treated with the preparation containing 2% maintained the number of fibroblasts at a higher level; its activity was better than the group treated with the preparation containing 5% and the group treated with phenytoin (common anti-seizure medication, here used for additional antibiotic properties) [74].

In another work, the authors prepared different extracts (using a variety of solvents, including n-hexane, diethyl ether, ethyl acetate and methanol) from the leaves of *S. ebulus*.

The extracts were embedded in an ointment base to obtain a preparation containing 1% of the extract. These preparations were applied on animal wound models characterized by linear incision (male Sprague–Dawley rats) and circular excision (male Swiss albino mice). In the linear incision wound model, the preparations, the reference compound (Madécassol^®^, containing 1% extract of *Centella asiatica*) and the vehicle were topically applied once in a day throughout nine days; while in the circular excision wound model, the preparations, the reference compound and the vehicle ointments were applied topically once a day till the wound was completely repaired. Preparations containing 1% methanol extract exerted a significant wound healing activity, the animals treated with this preparation showed 84.3% contraction in circular excision model (this percentage was closed to contraction value of the reference drug) and a significant increase (43.7%) in the wound tensile strength on incision wound model. Quercetin 3-*O*-glucoside was identified as one of the active compounds of the methanol extract [75].

### 3.8. Schisandra

Plants belonging to the genus *Schisandra* (fam. *Schisandraceae*) grow mainly in China, Japan, the Himalayas and Java, for a total of 25 accepted species. The parts traditionally used in medicine are seeds and fruits. *Schisandra chinensis* (Turcz.) Baill. (Chinese magnolia vine) is the best-known representative of the genus, widely used in the official East-Asian, European and North American medicine, especially for tonic and sedative effects. The main bioactive compounds are lignans; according to the European Pharmacopoeia the dried fruit should have a minimum schisandrin content of 0.40% [76].

#### 3.8.1. Skin Inflammation and Immunity

In literature, four studies (two In Vitro and two In Vivo) investigated the effects of *Schisandra* species on skin inflammation and immunity.

Guo et al. evaluated the ability of lignans from *S. chinensis*, called schisandrins A, B, and C, to inhibit some pro-inflammatory parameters in THP-1 cells infected by *Propionibacterium acnes*. The cells were pretreated with different concentrations of the molecules and infected with the pathogen. Schisandrin A, B, and C significantly inhibited IL-1β release at each concentration tested (5–10–20 µM), while for IL-8 release schisandrin A required a concentration of 10 μM to exert a significant effect. Schisandrin B and C, but not A, impaired TLR2 mRNA (10–20 µM), moreover TLR2 membrane expression was impaired by all the compounds (20 µM), with the following effect: Schisandrin B > C > A. The compounds possessed different selectivity for MAPKs: schisandrin C for ERK, schisandrin B for p38, schisandrin A for JNK. However, schisandrin C was able to significantly inhibit all MAPKs at 10 and 20 µM; moreover, p65 translocation was significantly reduced at 20 µM by all the compounds [77].

In another paper, the same research group investigated the effects of the compounds (10 µM) on *P. acnes*-induced NLRP3 inflammasome activation, in THP-1 cells, by measuring the levels of NLRP3, active caspase-1, mature IL-1β, and activity of caspase-1. Guo et al. demonstrated the ability of the three schisandrins to suppress *P. acnes*-induced pyroptosis in THP-1 cells; subsequently, the three lignans significantly suppressed all the parameters previously indicated (NLRP3 inflammasome activation), with the following potency: schisandrin C > schisandrin B > schisandrin A. These lignans significantly inhibited mitochondrial oxidation and ATP release [78].

Two In Vivo studies investigated the effects of *S. chinensis* on skin damage using a mice model of atopic dermatitis-like or contact dermatitis induced by 1-fluoro-2,4-dinitrofluorobenzene.

Jeon et al. demonstrated that the oral administration of the water extract from *S. chinensis* fruit (10–100 mg/kg) for 4 weeks after DNFB application, reduced the symptoms induced by the application of DNFB. The thickened skin and the infiltration of mast cells on skin tissue were reduced by *S. chinensis* extract compared to the group treated with DNFB only. The amount of cyanidin-3-rutinoside, the major anthocyanin of *S. chinensis* in the extract, was 3.48 mg/g. The authors investigated the effects of this compound on some inflammatory and allergy mediators in human mast cells (HMC-1) stimulated with PMA/A23187. Cyanidin-3-rutinoside, assessed in the range 0.1–100 µg/mL, was able to decrease IL-1β, TNF-α, and IL-6 secretion and gene expression induced by PMA/A23187; this compound inhibited PMA/A23187-induced phosphorylation of JNK, ERK, p38, and NF-κB (p65). The thymic stromal lymphopoietin (TSLP) mRNA levels, increased by PMA/A23187, were significantly reduced by treating the cells with 100 µg/mL cyanidin-3-rutinoside. PMA/A23187-stimulated histamine release was reduced by the compound in a concentration dependent fashion [79].

Lee et al. evaluated the anti-inflammatory activity of the methanol extract of *S. chinensis* (30–100–300 µg/ear), obtained from fruits, on DNFB-induced contact dermatitis in mice. Topical application of the extract daily for six consecutive days led to the inhibition of ear thickness (30 or 300 μg on the left ear, *p* < 0.001; 30 μg on the right ear, *p* < 0.001), hyperplasia, spongiosis (100 μg/ear, *p* < 0.05 and 300 μg/ear, *p* < 0.001, respectively) and immune cell infiltration (100 μg/ear, *p* < 0.05; 300 μg/ear, *p* < 0.001) induced by DNFB. Moreover, the extract reduced the production of some pro-inflammatory mediators, such as IFN-γ (significant effect starting from 30 µg/ear), TNF-α (significant effect starting from 100 µg/ear), IL-6 (significant effect at 30 µg/ear), and MCP-1 (significant effect starting from 30 µg/ear) [80].

#### 3.8.2. UV Damage

Four in vitro studies evaluated the activity of *Schisandra* species on skin injuries caused by UV exposure.

Hou et al. evaluated the effects of deoxyschisandrin and schisandrin B (100 μM), the two major lignans isolated from *S. chinensis*, in HaCaT cells exposed to UVB (30 mJ/cm^2^). The two compounds protected the cells from UVB-induced cell death and DNA damage. Furthermore, pretreatment with the two lignans, followed by UVB exposure, significantly reduced the ROS levels and cell apoptosis (−2.42% by deoxyschisandrin and −4.29% by schisandrin B). The authors also demonstrated the ability of deoxyschisandrin and schisandrin B to reduce the decrease of caspase-3, 8, and 9 levels and the increase of the corresponding cleaved forms, after UVB exposure, indicating the inactivation of apoptosis [81].

The ability of schisandrin B to counteract UVB damage was also tested in dermal fibroblasts and keratinocytes (HaCaT) by Gao et al. The cells were pre-treated with the compound (0.1–1–10 mM) and then exposed to UVB (30 mJ/cm^2^). In HaCaT cells, schisandrin B exerted an antioxidant activity decreasing the levels of intracellular ROS in concentration dependent manner, increasing the level of intracellular SOD activity, and reducing MDA levels, with the higher effect at the concentration of 10 mM. The authors showed that the COX-2, IL-6, and IL-18 gene expression were gradually reduced with increasing concentration of schisandrin B, after UVB irradiation. Gao et al. evaluated the effects of this compound on expression levels of MMP-1 mRNA and on the synthesis of type 1 pro-collagen after UVB exposure in fibroblasts; the results revealed that schisandrin B reduced MMP-1 gene expression and restored the synthesis of pro-collagen in concentration dependent way [82].

Guo et al. tested the activity of the *S. chinensis* fruit extract (2.5, 5, and 10 μg/mL) in HDF cells exposed to UVB (3000 mJ/cm^2^). MMP-1 secretion was significantly decreased in presence of *S. chinensis* fruit extract from 82.3% to 88.4% compared to only UVB irradiation group, while collagen was induced by 58.4%. Pretreatment with the extract exerted positive effects on the antioxidant system, in particular the reduction in the glutathione levels induced by UVB irradiation was reversed by the presence of *S. chinensis* extract (the effect was statistically significant starting from 5 μg/mL). The MDA levels, compared to UVB-irradiated control group, were significantly decreased following the pretreatment with the extract (10 μg/mL) [83].

The CO_2_ extract from *S. sphenanthera* fruits was assessed on HaCaT cells exposed to UVB (30 mJ/cm^2^). After irradiation, the cells were incubated with the non-polar *S. sphenanthera* extract (0.04–0.4–4–40 μg/mL) or indomethacin (0.04–0.4–4–40 μg/mL) for 24h. The extract inhibited PGE2 production, induced by UVB, starting from 4 μg/mL; the effect of the extract, at each concentration evaluated, was lower compared to the effect of indomethacin. UVB-induced COX-2 expression was significantly down-regulated in HaCaT cells by the *S. sphenanthera* extract at 40 μg/mL [84].

### 3.9. Vaccinium

*Vaccinium* (fam. *Ericaceae*) is a widely diffused genus of shrubs which preferentially grow in acid soils. Fruits are the part of the plant used for medicinal purposes, they are usually brightly colored, and most are edible to humans. Proanthocyanidins from *Vaccinium macrocarpon* Ait. (cranberry) and anthocyanins from *Vaccinium myrtillus* L. (bilberry) fruits are, respectively, used to treat chronic venous insufficiency and urinary tract infections. Other species studied for their possible cutaneous effects are *Vaccinium corymbosum* L. (highbush blueberry), *Vaccinium uliginosum* L. (bog bilberry), and *Vaccinium vitis-idaea* L. (lingonberry).

#### 3.9.1. Skin Inflammation and Immunity

Four studies—three in vivo studies and one clinical study—evaluated the effects of *Vaccinium* species on skin inflammation and immunity.

In a study conducted by Yamaura et al., the authors evaluated if a concentrated extract of *Vaccinum myrtillus* L. was able to alleviate pruritus in a mouse model of chronic allergic contact dermatitis. In male ICR mice and female BALB/c mice, acute pruritus was induced through substance P, while chronic allergic contact dermatitis by 2,4,6-trinitro-1-chlorobenzene (TNCB). During the experiment, the extract was orally administered, every day, at 400 mg/kg or 2000 mg/kg, while naloxone and dexamethasone were administrated at the dose of 1 mg/kg and 1.5 mg/kg, respectively. The extract did not show a reduction in substance P-induced scratching; however, it was able to decrease ear swelling in dose dependent way and suppress the increase in the number of scratching induced by TNCB. Both doses significantly suppressed the number of scratching, even if the dose of 400 mg/kg was more effective than the dose of 2000 mg/kg in the suppression of pruritus [85].

Kim evaluated the effects of oral administration of a mixture of polyphenols and anthocyanins derived from *V. uliginosum* on DNCB-induced atopic dermatitis in NC/Nga mice. Mice were treated with different doses of polyphenols plus anthocyanins (2.56 + 7.31, 5.12 + 14.62, 10.25 + 29.25 μg/kg of body weight), prednisolone (3 mg/kg of body weight) was used as reference compound. The results showed the ability of the mixture to reduce skin symptoms (total clinical severity score), clinical signs (such as ear thickness and scratching behaviors), epidermis thickness and the number of inflammatory cells, induced by the application of DNCB [86].

In another study, Kim et al. investigated the effects of an orally administration of a water extract from *V. uliginosum* on DNCB-induced atopic dermatitis in NC/Nga mice. The extract was administrated at 90–150–250 mg/kg of body weight. Prednisolone was used as positive control (3 mg/kg of body weight). *V. uliginosum* extract reduced atopic dermatitis-like skin lesions induced by DNCB, like ear thickness and the frequency of scratching episodes in a time-dependent manner. The extract also reduced epidermal thickening, the infiltration of eosinophils, mast cells, and degranulated mast cells induced by DNCB [87].

#### 3.9.2. UV Damage

Four in vitro studies investigated the effects of *Vaccinium* species on UV-induced skin damages.

Svobododa et al. evaluated the effects of a *V. myrtillus* fruit extract (anthocyanins 25% *w*/*w*; cyanidin and delphinidin derivatives were the most abundant) against UVA-induced skin damages in human keratinocytes (HaCaT cells). The cells were pre-treated with *V. myrtillus* (5–100 mg/L) before or after irradiation with UVA (10–40 J/cm^2^). Pre-treatment (1 h) or post-treatment (4 h) of HaCaT with fruit extract resulted in attenuation of UVA-caused damage. The extract was able to significantly reduce UVA-stimulated ROS formation, the percentage of inhibition, at the higher concentrations tested (50–100 mg/L) was around 55% in pre-treatment and around 50% in post-treatment. Both pre-treatment and post-treatment with the *V. myrtillus* fruit extract also inhibited UVA-caused lipid peroxidation, and blocked UVA-induced glutathione depletion in HaCaT cells [88].

Svobodova et al. also tested the ability of the *V. myrtillus* fruit extract to counteract UVB-induced skin damages in human keratinocytes (HaCaT cells). *V. myrtillus* (5–50 mg/L) was applied to the cells 1 h before UVB irradiation (200 mJ/cm^2^) or 4/8 h after UVB exposure. The extract was able to prevent and reduce DNA single strand break formation; in particular, during the post-treatment, the effect was maximal at the concentration of 10 mg/L (−70%). *V. myrtillus* reduced caspase-3 and 9 activity, two mediators involved in apoptotic cell death, and DNA fragmentation, an important hallmark of apoptotic process. This effect was exhibited in pre- and post-treatment. During post-treatment the RONS production was reported to control level (non-irradiated cells) at the concentrations of extracts of 25–50 mg/L. Treatment of HaCaT cells with *V. myrtillus* extract, before and after UVB irradiation, reduced IL-6 production [36].

Calò et al. studied the effects, on UV-induced injuries, of a water extract from *V. myrtillus,* in HaCaT cells. The cells were pre-treated for 1 h with extract at 320 μg/mL and then irradiated with UVA (8–40 J/cm^2^) or UVB (0.008–0.72 J/cm^2^) rays. The extract decreased the UVA- and UVB-induced oxidative stress, reducing UVA-induced ROS generation (but not UVB-induced ROS production), and lipid peroxidation caused by both UV rays, however the effect on this parameter was not statistically significant. The authors demonstrated the ability of the extract to significantly reduce UVA (16 J/cm^2^)-induced DNA damage, micronucleus formation after UVA (8 J/cm^2^), and UVB (0.008 J/cm^2^) irradiation. The extract showed anti-apoptotic effects after UVA exposure, but it was inactive on UVB-induced apoptosis [89].

Bae et al. investigated the anthocyanin-rich extract (ATH-BBe) from bog bilberry (*V. uliginosum*) in counteracting UVB-induced damages in human dermal fibroblasts. The cells were pretreated with ATH-BBe (1–10 mg/L) and exposed to 100 mJ/cm^2^ UVB. Bog bilberry extract reduced UVB-induced ROS production at 5 and 10 mg/L and blocked p53 and Bad phosphorylation, two proteins activated in response to DNA damage, at the concentration of 10 mg/L. Pre-treatment with ATH-BBe counteracted the reduction, caused by UVB rays, of procollagen, intracellular collagen and collagen secretion levels in concentration dependent way; moreover collagenolytic MMP secretion (MMP-1, MMP-8 and MMP-13), responsible for UVB-induced collagen degradation, was suppressed in concentration dependent manner, by pretreating the fibroblasts with ATH-BBe. The extract also showed the ability to diminish the UVB-induced amount of some secreted cytokines, such as IL-8, IL-6, TNF-α, and IL-1β. The authors demonstrated that ATH-BBe attenuated UVB-triggered dermal collagen destruction through blocking NF-κB activation and MAPK-signaling pathways (JNK and p38) [90].

#### 3.9.3. Wound Healing

According to the literature, two studies (one in vivo and one in vitro) investigated the activity of *Vaccinium* species on skin wound healing.

Nayak et al. evaluated the wound healing activity of cranberry oil (*V. macrocarpon*) by using an excision wound model in rats. Male Sprague–Dawley rats were treated topically with cranberry oil (100 mg/kg of body weight). The reference group was treated with mupirocin ointment (100 mg/kg of body weight), while control group was treated with petroleum jelly. At day 13, the animals treated with cranberry oil showed a reduction of the wound area of 88.1%, while the reference group exhibited a reduction of 78.4%. The hydroxyproline content of the granulation tissue was significantly higher in the animals treated with cranberry oil compared to the animals treated with petroleum jelly. The histological analysis of the granulation tissue of the cranberry oil-treated group showed a better organization of the bands of collagen compared to the control group. The amount of the phenolic derivatives in the cranberry oil, expressed as gallic-acid equivalents, was 3.167 ± 0.124 mg/g [91].

Esposito et al. prepared two polyphenol-enriched extracts, one from *V. uliginosum* (bog bilberry), and one from *V. vitis-idaea* (lingonberry), using acidified 70% aqueous methanol (0.5% acetic acid). Starting from the polyphenol-enriched extract of each varieties, they isolated an anthocyanin fraction and a proanthocyanidin fraction. The extracts, in particular bog bilberry extract and proanthocyanidin fractions, exerted anti-inflammatory and antioxidant effects in RAW 264.7 macrophages stimulated with LPS, reducing ROS and NO production and COX-2 and iNOS gene expression. Following, both polyphenol-enriched extracts (50 μg/mL) significantly increased cell migration in an exclusion zone-based wound healing model with primary human dermal fibroblasts isolated from adult skin (HDFa); among anthocyanin and proanthocyanidin fractions, the proanthocyanidin fraction from lingonberry was the most active on this parameter. The phytochemical analysis indicated that procyanidin B2 was a common component in the investigated berries; for this reason, the authors demonstrated that procyanidin B2 and bioactive metabolites (10 μM) could influence wound repair and tissue regeneration, stimulating mitochondrial bioenergetics and upregulating the expression of important extracellular matrix proteins in HDFa cells [92].

### 3.10. Vitis

The genus of *Vitis* (fam. *Vitaceae*) includes plants distributed mainly in temperate regions of the planet and consists of 79 accepted species. The species most cultivated and studied, *Vitis vinifera* L., is an important economical source of grapes, for wine production. According to the ESCOP monographs, the drug is constituted by the leaves of the cultivar “Teinturier,” used to treat varicosis and chronic venous insufficiency. Muscadine (*Vitis rotundifolia* Michx.) was the first American grape to be cultivated in the United States [93], and it has been studied for potential anticancer properties [94].

#### 3.10.1. Skin Inflammation and Immunity

In contrast with vascular context, grape seed extracts have never been investigated for the anti-inflammatory properties against skin disorders. Notably, the literature on the dermatological effects of *Vitis* spp.-derived extract is extremely poor, with only one in vitro and one in vivo study. Sangiovanni et al. demonstrated for the first time the anti-inflammatory activity of leaves water extract from *V. vinifera* L. in keratinocytes (HaCaT). The extract (1–50 μg/mL) inhibited IL-8 and VEGF release through NF-κB pathway, induced by TNF-α or LPS. The main phenols contained in leaves extract were quercetin glycosides and caftaric acid: they permeated ex vivo human skin (Franz cell test) ranging from 9 to 52 µg/g [95]. Similarly, Khanna et al. [96,97], who investigated proanthocyanidin-rich grapevine seed extract, demonstrated that VEGF release induced by H_2_O_2_ was inhibited. 

Bralley et al. [98] conducted an in vivo experiment with hydroalcoholic extract (50:50 water ethanol) from fruit skin or seeds of *V. rotundifolia* Michx. (muscadine). Swiss mice were treated topically with 2 µg/ear of single extracts or their combination after TPA-induced edema. Extracts of muscadine skin, seed, and combination treatments significantly reduced ear edema, ear biopsy weight, and MPO (−50%) activity. There was no significant difference in anti-inflammatory activity of the skin and seed extracts. However, an additive effect, comparable to indomethacin, was observed with the combination treatment regarding ear biopsy weight.

#### 3.10.2. UV Damage

The literature reports six papers—four in vivo and two in vitro—concerning the photoprotective activity of *V. vinifera* extracts. Grape seed proanthocyanidin–enriched extract (GSPE) has been evaluated in three out of the four in vivo studies. Sharma et al. [99] treated SKH-1 hairless mice, exposed to acute UVB dose (120 mJ/cm^2^) or to chronic UVB exposure (120 mJ/cm^2^, three times a week for one month), by oral administration (0.2% or 0.5%, *w*/*w* GSPE) with diet. The treatment inhibited the depletion of glutathione peroxidase, catalase, H_2_O_2_ production, lipid peroxidation, protein oxidation, and nitric oxide release in mouse skin after acute or chronic UV exposure. The authors observed also inhibition of ERK 1/2, c-Jun-NH2-kinase, and p38 phosphorylation, leading to impaired activation of NF-κB (p65) pathway. The effect reflected on cellular proliferation and pro-inflammatory enzymes expression, since GSPE inhibited the expression of PCNA, cyclin D1, iNOS, and COX-2 in the skin. The same authors extended the data regarding UVB-induced inflammation and carcinogenesis a few years later by the same in vivo model [100]. New insights on GSPE regarded the anti-proliferative effect and the anti-inflammatory mechanism. About the latter, leukocyte infiltration and MPO activity were impaired in the irradiated tissue; moreover, PGE2, TNF-α, IL-1β, and IL-6 levels were reduced.

Accordingly, Mittal et al. [101] evaluated the effect of GSPE (0.2 and/or 0.5%, *w*/*w*) in the same animal model (UVB-irradiated SKH-1 hairless mice). The author confirmed the preventive effect on photocarcinogenesis. Biochemical analysis revealed that treatment of GSPE significantly inhibited UVB- or Fe^3+^-induced lipid peroxidation. The long-term feeding with GSPE did not show apparent signs of toxicity in mice in terms of body weight and physical characteristics of internal body organs like spleen, liver, and kidney.

Che et al. [102] performed another in vivo study treating C57BL mice exposed to UVB irradiation (120 mJ/cm^2^) with stem cells extract. Topical treatment with extract significantly reduced erythema index and epidermal thickness. Levels of glutathione and MDA were recovered in the skin tissue, suggesting the involvement of antioxidant pathways; in fact, Nrf2 and HO-1 resulted down-regulated as well as in ascorbic acid-treated groups. The extract exerted also anti-inflammatory effect, since infiltration of mast cells and neutrophils COX-2 expression were significantly decreased. Moreover, collagen loss and MMP1 expression were prevented. 

Regarding in vitro testing, Marabini et al. [33] and Sangiovanni et al. [95] examined the effect of water extract from *V. vinifera* “Teinturier” leaves in UV-induced keratinocyte (HaCaT). The first paper reported the photoprotective activity of grapevine against UV-caused genotoxicity. Irradiation with UVA (10 and 15 J/cm^2^) caused a significant increase of phosphorylation of the histone protein H2AX in cells, that was reduced with the pre-treatment with *V. vinifera* at 100 µg/mL. Conversely, the result of Comet assays showed that UVB (40 mJ/cm^2^) damage was not prevented. Moreover, according to Sangiovanni et al. [95], water extract reduced the UVB induced IL-8 secretion at lower concentrations (IC_50_ 2.42 μg/mL) and NF-κB nuclear translocation at highest concentrations.

#### 3.10.3. Wound Healing

Grapevine has been studied in vitro (three papers) and in vivo (four papers) for wound healing properties. Among the total seven publications, five concerned grape seed proanthocyanidin-enriched extract (GSPE), one grape seed oil, and the last investigated grape skin powder. All the In Vitro studies were carried out with GSPE with a concentration range of 2.5–50 μg/mL. 

Sandra et al. [103] focused on the role of human fibroblasts (TIG 3-20) for remodeling. The mediators uPA (urokinase plasminogen activator) and PAI-1 (Plasminogen activator inhibitor-1) are antagonist for plasminogen conversion to plasmin. The latter participates to fibrinolysis and remodelling. Both mediators (mRNA and expression) were inhibited by GSPE extract at 20 μg/mL. The final effect resulted in impaired fibrinolytic activity. Moreover, GSPE decreased fibroblasts migration in imaging assay after scratch test. Interestingly, the outcome was the opposite of the expected one for wound healing mechanism, namely increase of migration and fibrinolysis. Since oxidation was considered a proliferative and pro-migratory effect, the antioxidant effect was purposed as mechanism for migration and fibrinolysis inhibition: in fact, basal intracellular ROS production was decreased by 80% at the same concentration.

Khanna et al. [96] investigated GSPE extract from the market, containing 54% dimeric, 13% trimeric, and 7% tetrameric proanthocyanidins, and 5000 ppm of trans-resveratrol. At 2.5–15 μg/mL, GSPE upregulated in a concentration-dependent manner VEGF mRNA and release when keratinocyte (HaCaT) were challenged by hydrogen peroxide or TNF-α, but not in the absence of pro-inflammatory mediators. This result was confirmed by a second study from the same authors [97]. The translation in vivo (Balb/C mice) showed complete healing of wound excision after the topical treatment with 25 μL of 100 mg/mL extract. The histological exam revealed increased epidermal keratin deposition, connective tissue deposition, and structure organization. This evidence correlated with the increase of VEGF release and oxidative markers (4-HNE; oxidized/reduced glutathione) in the tissue, thus confirming in vitro observations.

Another In Vivo study from Hemmati et al. [104] demonstrated the wound healing activity of GSPE applied topically in Eucerin base. Rabbits were treated twice a day with the cream (2–5–10–70%) after wound excision. All doses equally increased hydroxyproline content in the tissue. Moreover, GSPE 2% performed better than phenytoin in complete wound healing. The same author extended the evidence to a pilot clinical study, including 40 patients who underwent to surgery for skin tag removal; the application of 2% GSPE extract cream performed better than placebo in wound healing [105]. The result is sustained by another positive clinical study on caesarean section wound care, enrolling 129 women treated with standardized 5% GSPE ointment [106].

Nayak et al. [107] obtained the same evidence in vivo, but using grape skin powder, containing 0.250 g/100 g of total phenols and 0.131 g/100 g of anthocyanins. After wound excision in Sprague–Dawley rats, grape skin powder–based ointment (100 mg/kg) led to complete healing, which resulted comparable to mupirocin at the same dose. Once again, collagen deposition was increased, as previously reported by other authors for GSPE—the content in hydroxyproline in the tissue was superior in the treatment group than positive control mupirocin. The same author [91] investigated cold-pressed oil from grape seed in this model. The oil performed better than mupirocin for wound healing and increased hydroxyproline content. The characterization revealed omega-6 PUFA were the main components of the oil (68.6%), while omega 3 (0.4%) and phenols were a minor presence. 

## 4. Discussion

Acting as a fundamental interface between the human body and the environment, the skin is exposed to multiple triggers of inflammation. The relationship between berry administration and inflammatory response is actually the common thread of this review, which included three main inflammatory contexts, namely wounds, UV exposure, and multifactorial dermatitis with a strong immune component. Wound healing is a complex biological process, mainly characterized, at least in the acute phase, by the coagulation cascade, neutrophil and macrophage infiltration, and keratinocyte and fibroblast migration [108,109]. The contemporary pharmacological approach is based on the moist wound healing concept by which a wet medication may accelerate epidermal migration and prevent over-infections thanks to the barrier effect [110]. The mild antibacterial effect of berry extracts is accompanied by anti-inflammatory properties, thus suggesting that berries may be good candidates for the development of new wound treatments. However, the anti-bacterial properties have yet been explored by many publications and would need a specific revision work, which is not the actual purpose of this review. Several berries showed well-established traditional uses against skin injuries like burns and wounds, namely pomegranate (*P. granatum*) and elderberry (*S. nigra*), but their pharmacological validation is still needed [11,12,111]. This review has underlined In Vivo evidences of wound healing exerted by polar extracts of *E. oleracea*, *P. granatum*, *R. imperialis*, *Sambucus* spp., *V. macrocarpon,* and *V. vinifera*. Notably, most of the studies included positive controls with an antibacterial mechanism. In some cases, like *V. macrocarpon* and *V. vinifera*, the effect was demonstrated also for oil from seeds or seed extracts. *P. granatum* emerged as the most investigated berry, with 13 In Vivo published researches and one promising case-report. Dry polar extracts of peel or grinded peel from pomegranate have successfully been applied topically to wounds, with homogeneous doses among different works, ranging from 2 to 10%, often in ointments with a paraffin vehicle. The studies were mostly conducted on male Wistar rats, treated for 2–3 weeks immediately after wound excision, linear excision, or burn wound models. Unfortunately, the comparison among doses by mg/kg unit was not always possible because of the lack of clear reports about the amount of ointment applied on the skin. The dose is clearly reported in only two papers out of 13: 10 mg/kg of peel extract and 100 mg/kg of peel powder, published by Elzayat et al. [112] and Nayak et al. [113], respectively. Considering the average weight of Wistar rats (180 g) and a standard application of ointment (0.5 g) on the wounded area, it is possible to estimate a range of 28–280 mg/kg of extract in the other experiments. The poor characterization was a notable weak point of all the studies, since only four authors reported partial analytical data—Mo et al. [54], Lukiswanto et al. [52], and Yuniarti et al. [53] standardized the amount of ellagic acid, which was 13% for the first paper and 40% for the other two, while Hayouni et al. [55] carried out a qualitative characterization of the ellagitannins punicalagin A and B, ellagic acid and anthocyanins (delphinidin and pelargonidin glucosides). 

Differently from pomegranate, *V. vinifera* was poorly investigated for fruit and leave wound healing potential, but the well-known grape seed proanthocyanidin-enriched extract (GSPE) reached clinical application in two promising trials [105,106]. Conversely, only one and three in vivo studies were conducted on *S. nigra* leaves and *S. ebulus* fruit and leaves, respectively. 

An increase in collagen deposition, and fibroblast migration and proliferation were widely mentioned in histological observations and were common for *P. granatum* fruit, *S. ebulus* leaves, and GSPE from *V. vinifera*. Furthermore, this mechanism was demonstrated in vitro for *P. granatum*, but not for *S. ebulus* and GSPE extract. However, many other berries seem to act on remodeling through fibroblast stimulation, such as *E. oleracea*, *R. idaeus*, *R. imperialis*, and *V. uliginosum*. Another common histological observation was the re-organization of the wounded tissue and neo-vascularization after berry treatment. Several authors corroborated the histological examination with VEGF measurements, thus revealing the increase of this fundamental angiogenetic factor [23,53,97,114]. The role of specific cellular populations in boosting angiogenesis was not assessed, except for GSPE, which stimulated keratinocytes-derived VEGF in vitro [97]. The effect on remodeling may additionally involve the inhibition of metalloproteases, such as MMP-1 and MMP-2, as demonstrated for *E. oleracea*, *P. granatum,* and *V. uliginosum* extracts. In parallel, extracts from berries were able to dampen polymorphonucleate cell and mastocyte infiltration, thus reducing oxidative processes and inflammation through ROS, NO, IL-1β, and COX-2 impairment. 

Antioxidant and anti-inflammatory properties also explain the role of berries against UV-induced skin damage. UV exposure leads to oxidative stress and direct or indirect structural damages, such as DNA breakdown and mutations, lipid or protein oxidation, and the promotion of the inflammatory process. These molecular effects are responsible for acute and chronic effects like erythema, pigmentation, photoaging, and carcinogenesis [115]. Afaq et al. had already reviewed the literature (five papers) on the photoprotective effect of pomegranate extract in 2011, while the other berries have never been comprehensively reviewed before. *P. granatum*, *V. vinifera,* and *S. chinensis* were the most investigated, mainly through in vitro experiments. Little in vivo evidence—a total of eight studies for *P. granatum*, *R. nigrum*, *R. fruticosus*, *R. occidentalis,* and *V. vinifera*—were obtained in SKH-1 hairless mice exposed to UVB light ranging from 90 to 224 mJ/cm^2^. As expected, polar extracts were able to counteract edema and erythema formation through anti-inflammatory and antioxidant mechanisms. Secondly, the antioxidant effects were measured in terms of restoration of glutathione levels and decrease of ROS, MDA, and MPO in the irradiated tissue. The prevention of the DNA damage due to ROS production was reinforced by a direct photoprotective effect. Several papers reported a reduced occurrence of DNA damage markers (8-oxodG, CPD) and proliferative markers (PCNA, cyclin D1) after the treatment, which resulted in reduced carcinogenesis [68,100]. A controversial aspect emerging from several papers is the impact of berry extract on apoptosis as a preventive mechanism against photodamage. After the treatment with *Rubus* spp. berries, Duncan et al. [68] observed a lower p53 staining in UV irradiated tissues In Vivo, while Calvo-Castro et al. [34] observed an enhanced apoptosis in UV irradiated cells. The induction of the apoptotic machinery was also observed by Marabini et al. [33] and Syed et al. [48] in UV-induced keratinocyte treated with grapevine leave or pomegranate fruit extract. All these authors interpreted the pro-apoptotic effect as preventive for cancer development. On the contrary, experiments on *Vaccinium* spp. fruit extracts underlined anti-apoptotic effect against UV-induced cell death [89,90]. The consistent amount of In Vitro studies demonstrated similar photoprotective mechanisms, through antioxidant and anti-inflammatory effects on fibroblasts and keratinocytes, the first-line cellular populations exposed to UV damage. *E. oleracea*, *F.* × *ananassa*, *L. caerulea*, *R. adenotrichus*, *S. chinensis*, *P. granatum*, *V. myrtillus*, *V. uliginosum,* and *V. vinifera* have been investigated in vitro. Notably, *F.* × *ananassa* was directly applied to fibroblasts or used as physical filter against UVA irradiation, thus dissecting and demonstrating both the biological and the physical potential for photoprotection [30,32]. 

Multifactorial skin diseases are characterized by the strong participation of lymphocytes, with dysregulated immune function, eventually leading to a chronicization. The recent literature elucidated the key role of lymphocyte subsets in dermatological affections due to autoimmunity, such as atopic dermatitis and psoriasis. Since environmental aptens and autoantigens have been recognized as fundamental triggers in the etiopathology, the relationship between antigen presenting cells (namely, dendritic cells and keratinocytes) and lymphocytes is nowadays the focus of drug development (see references [40,108,116] on the topic). Indeed, new approaches of treatment are being investigated to counteract the increasing incidence of these skin disorders, but they still remain unsatisfactory for resistant dermatitis. Beside the use of FANS, corticosteroids, immunosuppressors (calcineurin-NFAT inhibitors like cyclosporin A and tacrolimus), and phototherapy, new drugs acting on the immune system have been recently developed, like anti-IL17 and anti-IL23 antibodies or PDE4 inhibitors (e.g., apremilast, crisaborole). 

Polar extracts from *P. granatum* L., *Ribes* spp., *S. chinensis*, *Vaccinium* spp., and *V. rotundifolia* berries have been applied in vivo to induced dermatitis. Notably, due to the small number of papers focusing on specific dermatitis phenotypes, inhomogeneous in vivo models were revised together. On the one hand, this approach was useful to identify common anti-inflammatory mechanisms, but on the other it represented a limit to underline scalable evidences. The more frequent way of administration was the oral one, with doses of extract ranging from 10 mg/kg to 200 mg/kg. The authors reported the reduction of skin thickness, dermatitis severity, and scratching behavior by polar extracts. Underneath the improvement of symptoms, there were decreased plasmatic and dermal levels of IgE, histamine, CD3+ lymphocyte and mast cell infiltration in tissue. Kim et al. [86] also measured a reduced transcription of CCL20 and eotaxin-1, both involved in atopic dermatitis. Moreover, together with Yamakura et al. [85] and Ashigai et al. [65], the same author showed lower levels of IL-4 in splenocytes and attributed part of the effect to the anthocyanin-rich fraction of *Vaccinium* spp. berries. Interestingly, *S. chinensis* berries polar extract has been assayed in a DNFB-induced dermatitis model with positive results after both topical or oral administration by Lee et al. [80] and Jeon et al. [79], respectively.

Unfortunately, only Graf et al. [117] performed an oral bioavailability study in rats, detecting the presence of malvidin-3-5-diglucoside and peonidin-3-5-diglucoside in plasma (nM) after consumption of *V. myrtillus* juice (15 mg/kg/day). In particular, the metabolites malvidin-3-glucoside and peonidin-3-glucoside were detectable in the gut lumen, especially in the ileum (around 100 nM/g), caecum, and colon (around 10 nM/g), thus suggesting the contribution of a microbiota-driven effect. On the other side Huston et al. [41] analyzed a skin stripping test revealing the trans epidermal passage of punicalagin at concentrations of 2.39 ± 0.29 nM/cm^2^, thus suggesting a possible interaction with deep skin cellular populations, like lymphocytes and dendritic cells. 

A few authors reported an intriguing inhibitory effect exerted by extracts from *F.* × *ananassa*, *P. granatum,* and *R. fasciculatum* on NFAT [28,118,119] using lymphoblasts or epidermal cells, but only punicalagin was further investigated as single compound. Notably, the impact of polyphenols on lymphocytes after topical or oral administration is partially unexplored and would require deeper investigations on pharmacokinetics. As an example, microbiota-derived metabolites from punicalagin [41] or other polyphenols should be considered for the investigation on circulating leukocytes. However, the systemic effects of berries on adaptive immunity is a fascinating and unexplored area of research. For example, urolithin A, an ellagitannin metabolite, dose-dependently inhibited (10-50 µM) proliferation and activation of CD4+, by calcium influx impairment through the STIM1/STIM2–Orai1–SOCE pathway [120], but its effect on dermatitis is still unknown. 

## 5. Conclusions

Despite the fact that a phytocomplex has pleiotropic effects by definition, the validation of specific targets is fundamental for the possible applications of berry extracts in clinical, nutritional or cosmetical contexts. Extracts from berries are mainly characterized by a pattern of polyphenols, such as anthocyanins, flavonols, and tannins, which seems to contribute to the biological activity. The mechanisms of action have been mostly investigated in fibroblasts or keratinocytes till now, thus supposing that all these molecules reach the above-mentioned cellular populations. On the contrary, skin bioavailability of bioactive compounds is rarely investigated and could supply key information to define the best way of administration. From in vivo studies, both topical and oral administration emerged as promising and deserve further investigations. However, the attribution of the bioactivity to specific polyphenols or their metabolites is still a grey area, and usually underlines a more potent effect of crude extracts than individual compounds. The standardization of extracts and subsequent human studies appear the two missing key points for the development of new plant-based approaches for skin disorders.

## Figures and Tables

**Table 1:** List of the berries plant species investigated at cutaneous level and included in this review.

Botanical Name		
*Euterpe oleracea* Mart. *Fragaria* × *ananassa* (Duchesne ex Weston) Duchesne ex Rozier *Lonicera caerulea* L. *Punica granatum* L. *Ribes fasciculatum* Siebold & Zucc. *Ribes nigrum* L.	*Rubus adenotrichus* Schltdl. *Rubus fruticosus* L. *Rubus idaeus* L. *Rubus imperialis* Cham. & Schltdl. *Rubus occidentalis* L. *Sambucus ebulus* L. *Sambucus nigra* L. *Schisandra chinensis* Baill.	*Schisandra sphenanthera* Rehder & E.H.Wilson *Vaccinium corymbosum* L. *Vaccinium macrocarpon* Ait. *Vaccinium myrtillus* L. *Vaccinium uliginosum* L. *Vaccinium vitis-idaea* L. *Vitis rotundifolia* Michx. *Vitis vinifera* L.

## Supplementary Materials

The following are available online at https://www.mdpi.com/2076-3921/9/6/542/s1, the acronyms used in the text and their meaning are collected in Table S1. List of acronyms.; the effects of plants producing berries in the healing of skin wounds, as well as the models in use, concentrations and reference compounds, are summarized in Table S2. Wound healing effects of plants producing berries [121,122].
