# Supplementary material for: A Review of the Potential Benefits of Plants Producing Berries in Skin Disorders
Source: Antioxidants (Basel). 2020 Jun 20;9(6):542. doi: 10.3390/antiox9060542 (PMC7346205; doi:10.3390/antiox9060542)
Supplement: Supplementary file 1 [file antioxidants-09-00542-s001.pdf]

Table S1. List of acronyms

Acronym	Definition	Acronym	Definition
4-HNE	4-hydroxy-2-nonenal	MAPK	mitogen-activated protein kinase
8-oxodG	8-Oxo-2'-deoxyguanosine	MCP-1	monocyte chemoattractant protein 1
Akt	protein kinase B	MDA	malondialdehyde
AMPK	5' AMP-activated protein kinase	MKK	mitogen-activated protein kinase kinase
AP-1	activator protein 1	MMP	metalloproteinases
BaPDE	benzoapyrene-diol-epoxide	MnSOD	manganese- dependent superoxide dismutase
CAT	catalase	MPO	myeloperoxidase
CD3	cluster of differentiation 3	mTOR	mammalian target of rapamycin
CD8	cluster of differentiation 8	MTT	(3-(4,5-dimethylthiazol-2-yl)-2,5-diphenyltetrazolium bromide
CoQ10	Coenzyme Q10	NF-κB	nuclear factor kappa B
CoQox	Coenzyme Q10 oxidized	NFAT	nuclear factor of activated T-cells
CoQred	Coenzyme Q10 reduced	NHEK	normal human epidermal keratinocytes
COX-2	cyclooxygenase 2	NLRP3	NLR Family Pyrin Domain Containing 3
CPD	cyclobutane pyrimidine dimers	Nrf-2	nuclear factor erythroid 2-related factor 2
DNCB	2,4-dinitrochlorobenzene	OCR	oxygen consumption rate
DNFB	1-fluoro-2,4-dinitrobenzene	p38	P38 mitogen-activated protein kinases
ERK	extracellular-signal-regulated kinase	PCNA	proliferating cell nuclear antigen
foxp3	forkhead box P3	PGE2	Prostaglandin E2
Gpx	glutathione peroxidase	PI3K	phosphoinositide 3-kinases
GSH	glutathione	PMA	phorbol 12-myristate 13-acetate
Gsta1	glutathione S-transferase 1	PMN	polymorphonuclear cells
H2AX	H2A histone family member X	PUFA	polyunsaturated fatty acids
HDF	human dermal fibroblasts	ROS	reactive oxygen species
HO-1	heme oxygenase 1	SOD	superoxide dismutase
IgE	immunoglobulin E	SPF10	sun protective formulation-10
IKBα	nuclear factor of kappa light polypeptide gene enhancer in B-cells inhibitor, alpha	STAT3	signal transducer and activator of transcription 3
IL	interleukin	TIMP	tissue inhibitor of metalloproteinases
iNOS	inducible nitric oxide synthase	TLR	Toll-like Receptor
JB6 Cl 41	epidermal mouse cells	TNCB	2,4,6-trinitro-1-chlorobenzene
JNK	c-Jun N-terminal kinase	TNF-α	tumor necrosis factor-α
LPS	lipopolysaccharide	UV	ultraviolet rays
LXR	liver X receptor	VEGF	vascular endothelial growth factor

Table S2. Wound healing effects of plants producing berries

Plant material/extraction	Evidence	Model/Assay	Dose and administration	Reference compound	Results (↑ increase; ↓ reduction)	Ref.
***Euterpe oleracea* Mart.**						
Water extracts from whole fruit powder (warm extraction, 80°C)	*In vitro*	HS68 fibroblast cells	0.1-1 mg/mL		↑ migration and proliferation; ↑ fibronectin<br>↓ MMP-1	[23]
		Enzymatic test	1-10 mg/mL	Ascorbic acid (1-10 mg/mL)	↓ elastase and collagenase	
	In vivo	Sprague Dawley rats, round excision	1-5% solution; topical appl	Ointment 20 mg/g (2%) of sodium fusidate	↓ wound area; ↓ mast cell infiltration<br>↑ collagen	
				//	↑ collagen, VEGF, fibronectin<br>↓ MMP- 1, IL-1β	
***Punica granatum* L.**						
Seed oil, fermented juice and water extract from fruit peel (warm extraction)	*In vitro*	Primary keratinocytes and fibroblasts	0,01-1 µl/mL	//	Seed oil: ↑ keratinocyte proliferation<br>Peel extract: ↑ fibroblasts proliferation, procollagen, MMP1	[58]
Powder from dry pomegranate rind	*In vivo*	Wistar albino rats, round excision	10% ointment	Fucidin ointment (fusidic acid)	↓ wound area vs positive control	[121]
Ethanol:water (3:1) extract from peel and pulp of rind	*In vivo*	Wistar rats, linear incision	Dose omitted, topical appl	Phenytoin	↓ wound area by peel extract, comparable to positive control<br>↑ epithelization, neo-vascularization, fibroblast count<br>↓ PMN and macrophage count	[122]
Commercial extract from peel	*In vivo*	Wistar rats, round excision	15% ointment, 10 mg/Kg, topically	Gentamycin 10 mg/Kg	↓ wound area	[112]
Methanol:water (3:1) extract from dried peel. Qualitative characterization of punicalagin A and B	*In vivo*	Guinea pigs, round excision	5% w/w ointment	2% cetrimide	↓ wound area ↑ total proteins, DNA, hydroxyproline<br>↑ collagen orientation and fibroblast proliferation<br>↓ macrophage infiltration	[55]
Extract from whole fruit (solvent omitted) titred for ellagic acid (40%)	*In vivo*	Albino rats, II-degree burn model (hot plate)	2.5-10% ointment	Silver sulphadiazine 1%	↓ wound area ↑ collagen deposition<br>↓ angiogenesis, PMN cells count	[52]
	In vivo	Wistar rats, linear incision			↑ tensile strength vs positive control;<br>Corresponding dose of EA (0.65%) showed comparable effect	[54]

Methanol:water (9:1) extract from peel dried. Ethyl acetate fraction titred for ellagic acid (13%).	*In vivo*	Wistar rats, round excision	2.5-10% ointment; corresponding 0,13%-0.65% ellagic acid	Xentella cream (7 % *Centella asiatica* extracts)	↓ wound area, inflammation: corresponding EA (0.65%) partially responsible<br>↑ hydroxyproline: EA not responsible	
	In vivo	Wistar rats, burn wound		Silver sulphadiazine 1%	↓ wound area; ↓ MPO. EA 0,65% partially responsible	
Ethanol:water (70:30) extract from flowers	*In vivo*	Wistar rats, burn wound	5-10% cream	Silver sulphadiazine 1%	↓ wound area vs positive control<br>↑ thickness, collagen orientation	[56]
Fruit peel powder	*In vivo*	Wistar rats, burn wound	100 mg/Kg of powder	Mupirocin 100 mg/Kg ointment	↓ wound area ↑ hydroxyproline vs positive control	[113]
Ethyl acetate extract from flowers	*In vivo*	Diabetic Wistar rats, round excision	0.2% ointment	Nitrofurazone (200 mg/Kg)	↓ wound area vs positive control; ↓ inflammatory infiltration<br>↑ in collagen	[57]
Ethanol extract from peel	*In vivo*	Diabetic Wistar rats, linear incision	Hydrogel 30% peel polyphenols (GA eq.)	//	↓ wound area; ↑ fibroblasts, collagen, vascularization<br>↑ hydroxyproline, NO, TGF-β1, VEGF, EGF	[114]
Extract from whole fruit (solvent omitted) titred for ellagic acid (40%)	*In vivo*	Wistar rats, linear incision	2.5-7.5% ointment	Betadine ointment	↑ collagen, neutrophil infiltration, angiogenesis, fibrosis	[53]
Ethanol:water (4:1) extract from peel powder titred for punicalagin and punicalin (22 mg/g as sum)	Human	A woman (76 years), chronic wound single case-study	2% hydrogel, topically	//	Complete healing after 12 weeks. Subject was not responsive to clostebol, neomycin, gentamicin, angiology care, silver sulphadiazine, betamethasone dipropionate	[59]
***Ribes nigrum* L.**						
Essential oil from buds, steam extraction	*In vivo*	Wistar rats, Burn wound	5% essential oil gel	Cicatrizin cream	↓ wound area	[61]
Methanol extract from leaves of *Ribes* spp.: *R. alpinum, R. anatolica, R. multiflorum, R. nigrum, R. orientale, R. petraeum, R. rubrum, R. uva-crispa*	*In vivo*	Swiss albino mice and Sprague-Dawley rats, linear excision	1% cream	Phyto Krem (containing 1% *Triticum vulgare* extract)	↓ wound area for *R. multiflorum* and *R. nigrum*: only *R. nigrum* comparable to positive control. Ethyl acetate fraction from *R. nigrum* more active then DCM and ButOH. The effect of EtAc fraction correlated with ABTS scavenging activity	[62]

***Rubus idaeus* L.**						
Liposoluble fraction of *in vitro* cultured cells from leaves (titred in linoleic and alpha-linolenic acid)	*In vitro*	Keratinocytes (HaCaT)	0.1% liposoluble fraction in medium	TO-901317 10 µM (LXR agonist)	↑ beta-glucocerebrosidase (comparable with positive control)	[70]
				Retinoic acid 1 µM	↑ expression of aquaporin-3, filaggrin, involucrin, hyaluronan synthase-3	
		HDF		Retinoic acid 1 µM or Ascorbate 300 µM	↑ expression of hyaluronan synthase-2, elastin, fibrillin I, lysil-oxidase;<br>↑ release of pro-collagen I, pro-collagen III, fibronectin	
	Human (n=20 women with dry or very dry skin)	Double blinded facial application, twice a day/28 days	0.1% liposoluble fraction	Moisturizing base cream	↑ hydration (+20%) vs moisturizing base cream	
***Rubus imperialis* Cham. & Schltdl.**						
Methanol extract from aereal part (leaves and branches) and respective n-hexane, chloroform, ethyl acetate fractions	*In vitro*	L929, horizontal scratch	1-100 µg/mL extract or 1-100 µM niga-ichigoside F1	//	↑ fibroblast migration by extract but not niga-ichigoside	[66]
		DPPH test	1-100 µg/mL	Ascorbic acid 50 µg/mL	Scavenging of DPPH (-75%) at 10 µg/mL	
	Ex vivo	Neutrophils from BALB/c mice after i. p. injection of oyster glycogen	1-100 µg/mL extract or 1-100 µM niga-ichigoside F1	//	Extract or niga-ichigoside: ↓ nitric oxide release (10 µg/mL and 10 µM); ↑ phagocytosis (10 µg/mL)	
	In vivo	Swiss mice, air pouch oedema	100 mg/kg	Indomethacin 30 mg/kg (pre-treatment)	↓ neutrophil infiltration, total leukocyte count	
		BALB/c mice, wound excision	1-2.5% in semisolid base	//	↓ wound area	
***Sambucus nigra* L.**						
Leaves tincture, 1:5 D/E (Romanian Pharmacopoeia X), 70% ethanol	*In vivo*	Wistar rats, burn wound	10% ointment	Silver sulphadiazine 1%	↓ wound area vs positive control	[72]

***Sambucus ebulus* L.**						
Methanol extract from fruit after percolation with hexane and ethyl acetate	*In vivo*	Wistar rats, wound excision	5% ointment	//	↓ wound area	[73]
Methanol:water (70:30) extract from leaves	*In vivo*	Wistar rats, wound excision	2-5% ointment	Phenytoin	↑ fibroblast count, neovascularization (2% ointment superior than positive control and 5% ointment)	[74]
n-hexane, diethyl ether, ethyl acetate, methanol subsequent extraction from leaves followed by bioguided fractionation of methanol extract	*In vivo*	Sprague-Dawley rats, linear incision	1% ointment	Madecassol (*C. asiatica* extract)	Methanol and ethyl acetate extract: ↑ tensile strength	[75]
		Swiss albino mice, round excision			Methanol extract: ↓ wound area (comparable with positive control); Ethyl acetate extract was less active	
		Chemical analysis			Quercetin-3-O-glu was the major compound in the most active fraction from methanol extract. The activity of the fraction was lower than the extract	
***Vaccinium macrocarpon* Ait.**						
Oil from seeds obtained by cold-pressing	*In vivo*	Sprague-Dawley rats, wound excision	100 mg/Kg ointment	Mupirocin 100 mg/Kg ointment	↓ wound area earlier vs positive control<br>↑ hydroxyproline	[91]
		Chemical analysis			3.167±0.124 mg/g GA eq. phenolic derivatives; 68% w/w of total PUFA; 31% w/w of omega-3 PUFA	
***Vaccinium uliginosum* L.**						
Polyphenol-enriched fractions from methanol:acidic water fruit extract (70:30, 0,5% acetic acid)	*In vitro*	Primary fibroblasts (HDFa)	50 μg/mL of extract or related anthocyanins and proanthocyanidins fractions	FBS 10% medium	↑ migration. Anthocyanin-enriched fraction but not PAC-enriched fraction was responsible	[92]
			10 μM of Procyanidin B2 or its structural subunits (HPCA, epicatechin)	//	↑ glycolysis by procyanidin B2, but not structural subunits; Structural subunits: ↑ oxygen consumption, ATP, ↓ proton leakage; In multigenic analysis: procyanidin B2 and subunits upregulated COL1A2 (pro-alpha2 chain of type I collagen), ITGB1 (integrin receptor subunit beta 1), and RHOA (ras homolog family member A);	

					Hypothesis: procyanidins modulate a complex interplay between ECM proteins (COL1A2 and ITGB1) and RHO guanosine triphosphate phosphatase (GTPase);<br>Down-regulated genes were MMP2 and CCL2	
		RAW 264.7 (mouse macrophages)	50 μg/mL of extract or related anthocyanins and proanthocyanidins fractions	Dexamethasone 10 μM	↓ ROS by fractions, but not extract;<br>↓ iNOS, NO, slightly COX-2 by extract and fractions;<br>Fractions were more active on COX-2	
		Chemical analysis			Total phenolics in phenol-enriched extracts (601±18 mg/g). The analysis showed also details on anthocyanins and proanthocyanidins (PAC) content	
***Vaccinium vitis-idaea* L.**						
Polyphenol-enriched fractions from methanol:acidic water fruit extract (70:30, 0,5% acetic acid)	*In vitro*	Primary fibroblasts (HDFa)	50 μg/mL of extract or related anthocyanins and proanthocyanidins fractions	FBS 10% medium	↑ migration. The anthocyanidin-enriched fraction and PAC-enriched were responsible for induced migration.	[92]
			10 μM of Procyanidine B2 or its structural subunits (HPCA, epicatechin)		[see *Vaccinium ulginosum* L.]	
		RAW 264.7 (mouse macrophages)	50 μg/mL of extract or related anthocyanins and proanthocyanidins fractions	Dexamethasone 10 μM	↓ ROS by fractions, but not extract, inhibited intracellular ROS; ↓ iNOS, NO, slightly COX-2 by extract and fractions	
		Chemical analysis			Total phenolics in phenol-enriched extracts (601±18 mg/g). The analysis showed also details on anthocyanins and proanthocyanidins (PAC) content	
***Vitis vinifera* L.**						
Proanthocyanidin-enriched fraction isolated from grape seed extract (ethanol:water 80:20)	*In vitro*	Human fibroblasts (TIG 3-20)	10-30 μg/mL	//	↓ ROS; ↓ uPA (urokinase plasminogen activator) and PAI-1 (Plasminogen activator inhibitor-1) antagonist for plasminogen conversion to plasmin;<br>↓ fibrinolytic activity, fibroblast migration<br>Hypothesis: anti-oxidant effect as mechanism for migration and fibrinolysis inhibition	[103]

Commercial grape seed proanthocyanidin extract; containing approximately 54% dimeric, 13% trimeric, 7% tetrameric proanthocyanidins, and 5000 ppm of trans-resveratrol	*In vitro*	Human keratinocytes (HaCaT), stimulation with H202	2.5-15 µg/mL	//	↑ VEGF vs H_2O_2 or TNF-α (at 15 µg/mL)	[96]
Commercial grape seed proanthocyanidin extract; containing approximately 54% dimeric, 13% trimeric, 7% tetrameric proanthocyanidins, and 5000 ppm of trans-resveratrol	*In vitro*	Human keratinocytes (HaCaT)	10 µg/mL	//	↑ VEGF promoter activity in absence or presence of H_2O_2	[97]
	In vivo	BalbC mice, wound excision	25 µL of 100 mg/mL extract	//	↓ wound area; ↑ keratin deposition, connective tissue, VEGF and oxidative markers (4-HNE; oxidized/reduced glutathione)	
Oil from seeds obtained by cold-pressing	*In vivo*	Sprague-Dawley rats, wound excision	100 mg/Kg	Mupirocin 100 mg/Kg ointment	↓ wound area vs positive control; ↑ hydroxyproline	[91]
		Chemical analysis			Phenolic derivatives: 3,330± 0.123 mg/g GA eq.; total PUFA: 68%; omega-3 PUFA: 0,4%	
Grape seed extract (80:20 ethanol:water)	*In vivo*	Rabbits, wound excision, treatment 2 per day/21 days	GSE 2-5-10-70% w/w Eucerin base	Phenytoin cream 1%	↓ wound area vs positive control by GSE 2%<br>↑ hydroxyproline at all doses	[104]
Grape skin dry powder (Cabernet Sauvignon variety)	*In vivo*	Sprague-Dawley rats, wound excision, once a day /13 days	100 mg/Kg ointment (vaseline base)	Mupirocin 100 mg/Kg ointment	↓ wound area, comparable with positive control<br>↑ hydroxyproline vs positive control	[107]
Grape seed extract (extraction and composition information omitted)	Human	40 patients after small surgey	2% cream	//	↓ wound area vs placebo	[105]
Grape seed extract (70:30 ethanol:water)	Human	129 women with caesarean section	5% ointment	//	Improvement of healing score (REEDA) vs placebo	[106]
